# DGAKI and PEI in dialogue 2023: Diagnostics and allergen immunotherapy 

**DOI:** 10.5414/ALX02450E

**Published:** 2023-12-12

**Authors:** Oliver Pfaar, Eckard Hamelmann, Christian Taube, Martin Wagenmann, Bettina Wedi, Thomas Werfel, Detlef Bartel, Andreas Bonertz, Diana Hartenstein, Susanne Kaul, Vera Mahler, Margitta Worm

**Affiliations:** 1 Section of Rhinology and Allergy, Department of Otorhinolaryngology, Head and Neck Surgery, University Hospital Marburg, Philipps-Universität Marburg, Marburg,; 2 Children’s Center Bethel, University Hospital OWL, Bielefeld University, Bielefeld,; 3 Department of Pulmonary Medicine, University Hospital Essen – Ruhrlandklinik, Essen,; 4 Department of Otorhinolaryngology, Düsseldorf University Hospital, Heinrich Heine University, Düsseldorf,; 5 Comprehensive Allergy Center, Department of Dermatology and Allergy, Hannover Medical School, Hannover,; 6 Department of Dermatology and Allergy, Hannover Medical School, Hannover,; 7 Paul-Ehrlich-Institut, Langen,; 8 Allergology and Immunology, Department of Dermatology, Venereology and Allergology, Charité –Universitätsmedizin Berlin, Berlin, Germany

**Keywords:** therapy allergen ordinance (TAO), guidelines, AIT products for children and adolescents, allergen diagnostics

## Abstract

A roundtable discussion on February 10, 2023 between the German Society for Allergology and Clinical Immunology (DGAKI) and the Paul-Ehrlich-Institut (PEI) aimed to discuss in detail current aspects of allergen immunotherapy (AIT), its regulatory framework under the transitional provision of the Therapy Allergen Ordinance (TAO), and the consequences for the planned guideline work of the DGAKI, regulatory challenges in the approval of AIT products for children and adolescents as well as allergy diagnostics. The content and discussion points of this dialogue are summarized and are set in context with the current literature.

## Topic 1: S2k guideline on allergen immunotherapy 2022 

The aim of the guideline published in September 2022 by the German, Swiss, and Austrian allergological societies and other specialist disciplines and professional associations was to formulate practical recommendations for the use of AIT as the only disease-modifying form of therapy for the treatment of allergic diseases based on the available evidence on the efficacy and safety of AIT. A total of 32 authors were involved in the guideline, with commentary and process support from representatives of the German Allergy and Asthma Association (DAAB) and PEI [1]. 

As a guideline in the Association of the Scientific Medical Societies in Germany (AWMF) Guideline Register, it was designed and created as an S2k guideline based on the AWMF Guidance Manual and Rules for Guideline Development [2]. Two important pillars make up the guideline. On the one hand, the guideline provides practical recommendations of different recommendation grades; on the other hand, the focus is on the requirement for a product-specific assessment of the evidence of AIT, as there is no generic class effect due to the biological starting materials and high product heterogeneity. In order to satisfy this aspect, the guideline authors have developed product-specific online tables that clearly display the differences between authorized AIT products and marketable AIT products subject to the TAO [3]. These tables are divided into three parts and provide the reader with an overview of a) their marketing authorization (MA) status in Germany in different age groups, b) the clinical documentation in published studies, and c) the current status of ongoing studies within the framework of the TAO process, insofar as these can be found in publicly accessible documents and platforms. 

This set of tables [4], which is updated every 6 months by delegates of the three German allergological societies, is currently unique in the world. 

The PEI is principally not in a position to pass on information about ongoing MA applications to third parties. Requirements for informing the public arise from Section 34 of the German Medicinal Products Act (AMG). Consequently, also the PEI occasionally refers to the information in the tables and the S2k guideline of the professional societies when receiving inquiries. 

During the discussion, representatives of the higher federal authority expressed their support of the evidence-based approach for the further development of the S2k guideline to S3 level. An update and a renewed systematic evaluation of the evidence, as last carried out in 2017 by the European Academy of Allergology and Clinical Immunology (EAACI) [5], would be of considerable importance for this. The aim should be to adequately record the current data from the study results that were stimulated by the TAO process and to specify practical recommendations on the efficacy and safety of AIT on this basis. Earlier meta-analyses, such as those published by the EAACI in 2017, also included study results from AIT products that are no longer on the German market – partly as a consequence of the TAO. It is anticipated that the evidence for the efficacy and safety of AIT will increase due to the increasing number of methodologically high-quality phase II and III studies on AIT included in a meta-analysis focused on currently authorized or marketable AIT products. It is expected that a further development of the guideline to S3 level, as discussed for 2027, could also increase the general perception of AIT with regard to disease management programs (DMP) on the basis of a systematic meta-analysis according to the latest methodological criteria. 

This could be done after completion of the TAO process, which is strongly supported by the DGAKI in order to continue to ensure the best possible treatment of patients with AIT. The last product-specific deadlines for AIT products under the transitional provision of the TAO end in 2026. Within the respective deadlines, pharmaceutical companies have the opportunity to collect the clinical data required for a MA in clinical trials in accordance with the current state of scientific knowledge and to complete their MA application dossier with these clinical trial data. The PEI then evaluates all submitted data in terms of quality, efficacy, safety and risk-benefit balance as part of the MA procedure. If the necessary data are not submitted within the granted period, further distribution of the AIT products under the transitional provision of the TAO is not possible and their marketability expires. However, further product development outside the TAO – without simultaneous marketing – with subsequent submission of a regular MA application once the product-specific study program has been completed is possible. 

A lively study activity within the above-mentioned deadlines can be observed. The decisions on MA applications could be directly incorporated into the planned guideline upgrade towards a possible (evidence-based) S3 guideline for AIT (Table 1). 

*Conclusion on topic 1 “AIT guideline”: Due to the increasing number of pivotal studies conducted according to the current state of scientific knowledge, the data situation on AIT will improve, and a subsequent, updated, and more precise systematic evidence analysis is considered useful. This could be carried out after completion of the assessments of the MA applications under the TAO, for which last product-specific deadlines for the submission of clinical data end in 2026. The DGAKI and PEI will remain in regular dialogue in this regard. A scenario with more comprehensive data could be a decisive argument for updating the AIT guideline to an S3 guideline. This would be an important step towards the development of a “national allergy action plan”.*

## Topic 2: Therapy allergen ordinance – current status of marketing authorizations 

The TAO came into force in 2008 [3]. To date, following further development, two of the AIT products originally subject to TAO have received MA in their new form. At the time of the DGAKI-PEI discussion (as of February 2023), 44 MA applications are still active. An overview of authorized AIT products and marketable therapy allergens under the transitional provision of the TAO can be found on the corresponding PEI website [6]. 

The transitional provision of the TAO stipulates that the deadline of 1 year for the submission of clinical data can be extended by a maximum of 7 years if this is necessary to remedy inadequate clinical data due to the nature of therapy allergens. A part of these 7 years was initially set as the deadline for the submission of data from dose-finding studies and a decision of a deadline extension was only made once these data had been submitted. The last product-specific deadlines end in 2026 (Table 1). After approval of an application to conduct a clinical trial, the respective studies can be started, and 1 year after their completion, the study results must be published on the study portals of the European Medicines Agency (EMA) [7]. However, there is no legal obligation to conduct an approved clinical trial. 

From the point of view of DGAKI, the requirement for convincing results in dose-finding studies as a basis for subsequent phase III studies in exertion of TAO [3] is well founded and represents a central pillar of the evidence-based assessment of AIT. In the study programs, clinical trials should be conducted according to the current state of scientific knowledge, and, for example, established and well-founded endpoints should be selected to demonstrate the clinically relevant efficacy of AIT products. Accordingly, the primary endpoint for allergic rhinitis/rhinoconjunctivitis in pivotal studies should be a combined symptom and medication score (CSMS), e.g., as published by the EAACI [8]. Other definitions for such combined scores can also be accepted as primary endpoints, provided they are scientifically justified. Numerous other endpoints can be evaluated as secondary endpoints. For a positive assessment of clinical trial data, there must be a statistically significant positive result in the primary endpoint. For MA, this result must also show a clinically relevant effect, i.e., the difference between the active treatment and placebo must be of a magnitude that is perceived by the patient as a meaningful and relevant improvement. In individual cases, the overall assessment of all primary and secondary endpoints can also lead to confirmation of clinical relevance. 

*Conclusion on topic 2 “Current status of the TAO”: The TAO, which came into force in 2008, aims to transition AIT products that are marketed as named patient products and contain active ingredients from sweet grasses (excluding maize), birch, alder, hazel, house dust mites, bee venom, and/or wasp venom into authorized products with confirmed quality, efficacy, and safety. The last product-specific deadlines for submitting the respective data under the transitional provision of the TAO end in 2026. If the clinical deficiencies are not resolved by the end of this deadline, the MA will be declined by the higher federal authority and the AIT product may no longer be marketed. If the available study results do not indicate sufficient efficacy and safety, the higher federal authority will no longer grant batch release during the ongoing TAO process so that the corresponding products can still be developed under the TAO, but no new batches will be placed on the market. This has already been carried out for a number of AIT products.*

## Topic 3: Pivotal studies in children and adolescents 

As with other diseases, there are still too few authorized products for the treatment of allergies in children and adolescents, which indicates the urgent need for further clinical studies and MA in this age group. As part of the Paediatric Regulation (Regulation (EC) No. 1901/2006), the Paediatric Committee (PDCO [9]) was established at the European level. For AIT products, there is the “*EMA/PDCO Standard Paediatric Investigation Plan for Allergen Products for Specific Immunotherapy*”*, *which defines the regulatory framework for pediatric investigation plans (PIP). The PDCO considers AIT to be indicated for children only with the aim of disease modification. Therefore, in the PIP for each product, one clinical trial in children must first be planned to collect long-term data to demonstrate a disease-modifying effect. However, each manufacturer must only carry out one long-term study in adults and – with a certain time lag – in children for a single product (the “selected product”) of the company’s portfolio in order to create a scientific basis for a possible extrapolation of data from adults to children. After conducting the studies with this selected product, the planned long-term studies for all other products can be changed to short-term studies in the respective PIPs of this manufacturer. However, the feasibility of long-term placebo-controlled studies in children has been the subject of controversy for years. 

Due to the vulnerability of this patient group, special requirements regarding risk and burden must be met for the approval of clinical trials in children and adolescents, which is why study results on the efficacy of the product from an adult study population are generally required before a trial in children is approved. A clinical trial in a pediatric population without available data from a trial in adults requires justification (e.g., no sufficient trial results expected in adults). In addition to the studies required by the PDCO to collect long-term data, for all “non-selected products” manufacturers are free to conduct clinical trials in children for short-term efficacy beforehand (as long as PIP compliance exists through deferral) in order to apply for an indication for the treatment of allergic symptoms. 

At present, there is only a limited number of products with an MA for children and adolescents that can be used by physicians. In addition, there are some products in the TAO process which, according to the product information, may be used in children; however, these are not authorized but currently marketed and prescribed as named patient products. Physicians are concerned that the lack of MA will sooner or later lead to the risk of recourse by health insurers. Some doctors are temporarily using AIT products that are only authorized for adults as off-label use in children and adolescents until they are authorized for this age group. 

There is a need to extend the indications for use in children and adolescents. However, further stipulations for studies in children are determined by the PDCO of the EMA and not by PEI. The way forward preferred by the manufacturers would be to align the regulatory requirements in the PIPs regarding AIT studies in children with those for MA in adults, i.e., to accept the successful treatment of allergic symptoms in 1-year studies as a reasonable therapeutic goal. This indication is currently not accepted by the PDCO. It is therefore necessary to develop alternative study designs for the investigation of AIT products in children (Table 1). Addressing this professionally and therapeutically relevant dilemma politically was identified as an important objective by the specialist associations in the roundtable discussion. 

*Conclusion on topic 3 “Problem of pivotal studies in children and adolescents”: The PDCO of the EMA requests long-term data for AIT products in these age groups to demonstrate disease modification. Before conducting studies in children, data on safety and efficacy in adult patients must be collected to avoid exposing minors to unnecessary risk.*

*To date, there are only a few AIT products with corresponding data for children and adolescents, which severely limits the scope of application of AIT in this age group with authorized products. An urgent goal for the optimal treatment of children and adolescents with allergic diseases in the future will be to set more realistic requirements for the evidence required for AIT products. To this end, it will be important to design meaningful studies that can be implemented in order to meet the right of children and adolescents to evidence-based therapy.*

## Topic 4: Marketing authorization of allergy diagnostics 

There is an increasing decline in the availability of allergen extracts for in vivo allergy diagnostics in Germany, e.g., for skin tests or allergen provocation tests. Manufacturers are returning existing MA for rare test allergens. The reason given for this is their economic inefficiency, as manufacturers must fulfill the requirements of good manufacturing practice and quality standards also for rare test allergens in accordance with the corresponding MA. This is associated with costs. Thus, according to the pharmaceutical companies, if demand for test allergens is low, they are unprofitable, with the consequence that their product portfolio is significantly reduced; consequently, the availability of basic allergy diagnostics is increasingly impaired (Table 1). 

There was a consensus among all those involved that ways must be sought to halt this downward spiral. Since 2018, the PEI has granted a 75% reduction in fees to 25% for all official procedures in connection with rare test allergens upon informal application in order to support the availability of test substances. With regard to rare test allergens, an extension of authorizations to other EU member states within the framework of Mutual Recognition Procedures could possibly increase profitability. However, this only seems realistic if a fee reduction for test allergens were also granted at European level, i.e., in all member states. This also applies to EMA pharmacovigilance fees. Support from politicians in dialogue with the allergological societies, professional associations, and manufacturers is required in this matter. Another possible solution could be the pharmacy production of test allergens. However, this requires the availability of certified starting materials and highly qualified and specialized pharmacies with additional equipment, which could produce test solutions as required – without MA – however taking into account the German Medicinal Products Act (AMG), European Pharmacopoeia, and the Pharmacy Operating Ordinance. As part of a scientific investigation [10] at the PEI, the feasibility and limitations of such potential pharmacy production of rare test allergens are currently being explored using the example of 20 test allergens for the identification of occupational allergies. 

*Conclusion on topic 4 “Marketing authorization of allergy diagnostics”: The portfolio of test allergens offered for allergy diagnostics is continuously decreasing. According to the manufacturers, this is due to market economy aspects, which make rarer allergen sources appear unattractive due to the unfavorable ratio of costs and revenue opportunities. Solutions are to be developed in close cooperation between authorities, professional associations, industry, and politics in order to maintain allergy diagnostics as the cornerstone of optimal treatment for allergy patients.*

In summary, the aim of the “Allergen Immunotherapy 2023 Dialogue” was to discuss current and important aspects and pressing issues relating to AIT and allergy diagnostics (Table 1). It was particularly important to the representatives of the DGAKI and the PEI to emphasize that optimal future patient care is and will remain the primary goal of all parties involved and that this should be achieved through regular joint dialogues. This goal will continue to be supported by the development of the “National Allergy Action Plan” by the DGAKI together with other specialist societies. 

## Funding 

The authors report no funding. 

## Conflict of interest 

O. Pfaar declares: Grants and/or personal fees and/or travel-support from ALK-Abelló, Allergopharma, Stallergenes Greer, HAL Allergy Holding B.V./HAL Allergie GmbH, Bencard Allergie GmbH/Allergy Therapeutics, Lofarma, ASIT Biotech Tools S.A., Laboratorios LETI/LETI Pharma, GlaxoSmithKline, ROXALL Medizin, Novartis, Sanofi-Aventis und Sanofi-Genzyme, Med Update Europe GmbH, streamedup! GmbH, Pohl-Boskamp, Inmunotek S.L., John Wiley and Sons, AS, Paul-Martini-Stiftung (PMS), Regeneron Pharmaceuticals Inc., RG Aerztefortbildung, Institut für Disease Management, Springer GmbH, AstraZeneca, IQVIA Commercial, Ingress Health, Wort&Bild Verlag, Verlag ME, Procter&Gamble, ALTAMIRA, Meinhardt Congress GmbH, Deutsche Forschungsgemeinschaft, Thieme, Deutsche AllergieLiga e.V., AeDA, Alfried-Krupp Krankenhaus, Red Maple Trials Inc., Königlich Dänisches Generalkonsulat, Medizinische Hochschule Hannover, ECM Expro&Conference Management, Technische Universität Dresden, Lilly, Paul Ehrlich Institut (PEI), FoMF GmBH, all outside the submitted work and within the last 36 months; and he is member of EAACI Excom, member of ext. board of directors DGAKI; coordinator, main- or co-author of different position papers and guidelines in rhinology, allergology and allergen-immunotherapy. 

E. Hamelmann declares: Research funding from the Bundesministeriums für Bildung und Forschung (BMBF) and the Landesministerien für Gesundheit (LMG) and for Forschung (LMF) of Nordrhein-Westfalen. He received honorarium for counseling from AImmune, ALK, Astra, GSK und SANOFI. 

C. Taube declares: No conflict of interest. 

M. Wagenmann declares: Lectures and adboards: ALK-Abelló, AstraZeneca, CSL Behring, Genzyme, GSK, HAL Allergie, Infectopharm, LETI Pharma, Novartis, Regeneron, Sanofi, Stallergenes; Research funding: ALK-Abelló, AstraZeneca, EU, GlaxoSmithKline, Novartis, Regeneron, Sanofi-Aventis, Takeda. 

B. Wedi declares: Honoraria for presentations, half-day advisory boards or travel/congress support from ALK-Abéllo, Bencard, Biocryst, CSL-Behring, HAL-Allergy, Novartis, Sanofi-Aventis, Takeda, ThermoFisherScientific. 

T. Werfel declares: grants from AbbVie, Beiersdorf, LEO, Novartis, Vortragstätigkeit: AbbVie, Allmiral, Galderma, Janssen, LEO, Lilly, Mylan/Viatris, Novartis, Pfizer, Sanofi/Regeneron; honorarium from Abbvie, Allmiral, LEO, Lilly, Mylan/Viatris, Novartis, Pfizer, Regeneron/Sanofi. 

D. Bartel declares: No conflict of interest.* 

A.Bonertz declares: No conflict of interest*. 

D. Hartenstein declares: No conflict of interest*. 

S. Kaul declares: No conflict of interest*. 

V. Mahler declares: No conflict of interest*. 

M. Worm declares: Receipt of honoraria or consultation fees by the following companies: Novartis Pharma GmbH, Sanofi-Aventis Deutschland GmbH, DBV Technologies S.A, Aimmune Therapeutics UK Limited, Regeneron Pharmaceuticals, Inc, Leo Pharma GmbH, AstraZenceca GmbH, ALK-Abelló Arzneimittel GmbH, Lilly Deutschland GmbH, Kymab Limited, Amgen GmbH, Abbvie eutschland GmbH & Co. KG, Pfizer Pharma GmbH, Mylan Germany GmbH (A Viatris Company), AstraZeneca GmbH, Lilly Deutschland GmbH, Boehringer Ingelheim Pharma GmbH & Co. KG, GlaxoSmithKline GmbH & Co. KG and FomF GmbH. 

*The views expressed in this paper are the personal views of the author as an expert in the field of allergology and may not be understood or quoted as being made on behalf of or reflecting the position of the respective national competent authority, the European Medicines Agency, or one of its committees or working parties. 

**Table 1.** DGAKI-PEI dialogue: Allergen immunotherapy (AIT) 2023 – key messages and next steps.

**Topics**	**Key message**	**Necessary further steps**
S2k AIT guideline 2022	The current literature contains an increasing number of methodologically high-quality studies on AIT. The growing evidence for the efficacy and safety of AIT will enable the current AIT guidelines to be updated to S3 level in the medium and long term.	The increasing number of study results makes it possible to re-evaluate the AIT evidence through systematic meta-analyses to further develop the AIT guideline to S3 level. This will be an important step towards a “National Allergy Action Plan”.
TAO – current status of the MA process	The TAO, which came into force in 2008, aims to transfer AIT products containing active ingredients from sweet grasses (excluding maize), birch, alder, hazel, house dust mites, bee venom, and/or wasp venom from existing named patient products into products with MA of proven quality, efficacy, and safety. For this purpose, clinical development programs with studies of appropriate methodological quality are carried out under the transitional provision of the TAO, the results of which must be available by 2026 at the latest in order to be included in the assessment by the higher federal authority (PEI).	The data required for the approval of AIT products according to the modern methodological criteria of the TAO must be available by the end of the product-specific deadlines under the TAO at the latest; incomplete MA dossiers will result in the decline of a MA and loss of marketability in Germany. If there is no proof of clinical efficacy or if there are safety concerns, market access is stopped already during the ongoing TAO process before the maximum extension is granted or by refusing further batch releases.
Challenges of pivotal studies in children and adolescents	The PDCO of the EMA has provided a standard PIP after consultation with the manufacturers. According to this standard PIP, manufacturers are obliged to plan a long-term study in children for each product and to carry out a long-term study in adults and children for at least one product in their portfolio in order to create a data basis for the long-term efficacy of the AIT and to be able to extrapolate data from adults to children in the future if necessary. However, only a few clinical trials are currently being conducted in children and adolescents. The feasibility of long-term placebo-controlled studies in children has been the subject of controversial debate for years.	An important goal for the use of AIT in children and adolescents is to have authorized products for which the indication of treatment in children is confirmed by the results of clinical trials in children and adolescents. To this end, alternative, feasible, and realistic study designs must be developed for this age group.
MA of allergy diagnostics	The portfolio of test allergens offered for allergy diagnostics is continuously decreasing. The reason given by the manufacturers is an imbalance between the costs of producing test allergens from rare allergen sources and the potential revenue from them.	The manufacturing companies, authorities, allergology associations, doctors, and political decision-makers are called upon to develop a joint concept to prevent a possible undersupply of allergy diagnostics.

AIT = allergen immunotherapy; DGAKI = German Society for Allergology and Clinical Immunology; EMA = European Medicines Agency; MA = marketing authorization; PDCO = Pediatric Committee; PEI = Paul-Ehrlich-Institut; PIP = pediatric investigation plan; TAO = Therapy Allergen Ordinance.
